# Dupilumab suppresses type 2 inflammatory biomarkers across multiple atopic, allergic diseases

**DOI:** 10.1111/cea.13954

**Published:** 2021-06-26

**Authors:** Jennifer D. Hamilton, Sivan Harel, Brian N. Swanson, William Brian, Zhen Chen, Megan S. Rice, Nikhil Amin, Marius Ardeleanu, Allen Radin, Brad Shumel, Marcella Ruddy, Naimish Patel, Gianluca Pirozzi, Leda Mannent, Neil M. H. Graham

**Affiliations:** ^1^ Regeneron Pharmaceuticals, Inc Tarrytown NY USA; ^2^ Sanofi Bridgewater NJ USA; ^3^ Sanofi Cambridge MA USA; ^4^ Sanofi Chilly‐Mazarin France

**Keywords:** asthma, atopic dermatitis, chronic rhinosinusitis with nasal polyposis, dupilumab, eosinophilic esophagitis

## Abstract

**Background:**

Type 2 inflammation is common in numerous atopic/allergic diseases and can be identified by elevated biomarker levels. Dupilumab, a fully human monoclonal antibody, blocks the shared receptor component for interleukin‐4 and interleukin‐13, key and central drivers of type 2 inflammation.

**Objective:**

Assessment of dupilumab effect on type 2 inflammatory biomarkers in atopic dermatitis (AD), asthma, chronic rhinosinusitis with nasal polyps (CRSwNP) and eosinophilic esophagitis (EoE).

**Methods:**

Data were extracted from three randomized placebo‐controlled trials of dupilumab in AD (NCT02277743, *N = 671*; NCT02277769, *N* = 708; NCT02260986, *N* = 740); and one each in asthma (NCT02414854, *N* = 1902); CRSwNP (NCT02898454, *N* = 448); and EoE (NCT02379052, *N* = 47). Biomarkers assessed were serum thymus and activation‐regulated chemokine (TARC), plasma eotaxin‐3, serum total immunoglobulin E (IgE), serum periostin and blood eosinophil count.

**Results:**

Dupilumab versus placebo significantly suppressed most type 2 inflammatory biomarker levels across all studies/indications where data were assessed. Reductions in serum TARC, plasma eotaxin‐3 and serum periostin occurred rapidly, whereas reductions in serum total IgE were more gradual. Across diseases, at the end of treatment, median percentage change from baseline in TARC levels ranged from −24.8% to −88.6% (placebo +2.6% to −53.6%); −38.2% to −51.5% (placebo +8.3% to −0.16%) in eotaxin‐3; −24.8% to −76.7% (placebo +8.3% to −4.4%) in total IgE; and −13.6% to −41.1% (placebo +10.1% to −6.94%) in periostin levels. Blood eosinophil responses to dupilumab varied by disease, with minimal changes in AD in the SOLO studies (median percentage change from baseline to end of treatment: 0% [95% CI: −15.8, 0]); transient increases followed by decreases to below‐baseline levels in asthma (−14.6% [−20.0, −7.7]) and CRSwNP (−29.4% [−40.0, −16.3]); and significant decreases in EoE (−50.0% [−50.0, −33.3]).

**Conclusion and clinical relevance:**

Dupilumab reduced levels of type 2 biomarkers across clinical studies in patients with AD, asthma, CRSwNP and EoE.

## Graphical abstract

This analysis assessed dupilumab effect on biomarkers of type 2 inflammation in atopic dermatitis, asthma, chronic rhinosinusitis with nasal polyps, and eosinophilic esophagitis. Dupilumab vs. placebo suppressed thymus and activation‐regulated chemokine, total immunoglobulin E, periostin, and eotaxin‐3 in all studies/indications where assessed; blood eosinophil responses varied by disease. Dupilumab may affect a shared pathophysiological mechanism fundamental to type 2 inflammatory diseases.

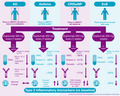

**KEY MESSAGES**

- This analysis assessed dupilumab effect on biomarkers of type 2 inflammation in AD, asthma, CRSwNP and EoE.
- Dupilumab suppressed TARC, total IgE, periostin and eotaxin‐3 in all studies/indications; blood eosinophil responses varied.
- Dupilumab may affect a shared pathophysiological mechanism fundamental to type 2 inflammatory diseases.

## 1 INTRODUCTION

**FIGURE 1:**
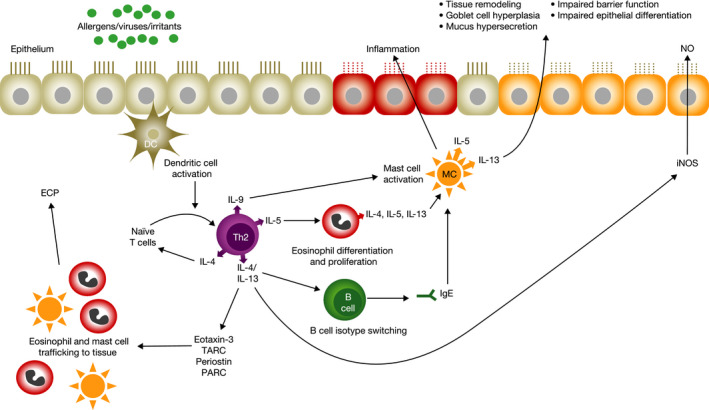
The roles of biomarkers in type 2 inflammation. DC, dendritic cell; IL, interleukin; ECP, eosinophil cationic protein; iNOS, inducible nitric oxide synthase; PARC, pulmonary and activation‐regulated chemokine; TARC, thymus and activation‐regulated chemokine

Type 2 inflammation underpins the pathophysiology of multiple atopic/allergic diseases that often have diverse clinical manifestations involving various organ systems, including atopic dermatitis (AD), severe asthma, chronic rhinosinusitis with nasal polyps (CRSwNP),^1^ and eosinophilic esophagitis (EoE).^2^ Involving both innate and adaptive immune systems, type 2 inflammation is characterized by the release of cytokines such as interleukin (IL)‐4, IL‐13, and IL‐5, resulting in tissue infiltration by eosinophils, mast cells and basophils, excessive mucus production in the airway, epithelial hyperplasia, tissue remodelling and allergy association. These changes are accompanied by debilitating symptoms, greater use of corticosteroids and other immunosuppressants, suboptimal disease control and poor quality of life.^3^, ^4^ Several biomarkers have been linked to type 2 inflammation (Figure 1), including serum thymus and activation‐regulated chemokine (TARC or CC chemokine ligand 17 [CCL17]), plasma eotaxin‐3 (CCL26), serum total immunoglobulin E (IgE), serum periostin, fractional exhaled nitric oxide (FeNO) and blood/tissue eosinophil count. These markers were selected because they are known to be regulated by IL‐4 and/or IL‐13 and are associated with various pathological features of type 2 inflammatory diseases. TARC and eotaxin‐3 are key chemokines for attracting inflammatory cells into the target tissue, including Th2 lymphocytes and eosinophils.^5^, ^6^, ^7^ Periostin is an extracellular matrix protein associated with activated fibroblasts, thought to play a role in the tissue remodelling and fibrosis observed in atopic diseases.^8^ IL‐4 and IL‐13 signal via transcription factors such as STAT6, inducing the downstream expression of TARC, eotaxin‐3 and periostin. In the STAT6 pathway, IL‐4 binds to IL‐4α and the IL‐4/IL‐4Ra complex recruits and pairs with either IL‐2Ryc or IL13Ra1 in heterodimeric receptors. Binding of the complex to type yc chain forms type I receptors, common in lymphocytes, whereas both IL‐4 and IL‐13 bind the shared type II receptor complex, containing IL13Ra1.^9^ Activation of Janus kinases results in generation of phosphorylated STAT6 which in turn up‐regulates genes that govern production of mediators of inflammation. Since the distribution of type I and II receptor expression varies among cells types, IL‐4/IL‐13 have differential effects in tissues. IL‐4 is also required for immunoglobulin isotype switching to IgE, a key downstream mediator in the type 2 adaptive immune response.^10^ Nitric oxide, a ubiquitous messenger molecule, is also a key inflammatory mediator in the respiratory tract whose production is partly regulated by IL‐13. Nitric oxide is produced by several cell types, including epithelial cells, mast cells, macrophages, neutrophils and vascular endothelial cells.^11^ Eosinophils are pro‐inflammatory leukocytes recruited from the bloodstream to inflammation sites by IL‐5 and the eotaxin family of chemokines.^5^ Eosinophilia and IgE production are hallmarks of type 2 inflammation in asthma, AD and CRSwNP,^1^, ^10^ and type 2 activation results in eosinophilia and other features of EoE.^12^

Dupilumab, a fully human monoclonal antibody,^13^, ^14^ blocks the shared IL‐4Rα component of IL‐4 and IL‐13 receptors, thus inhibiting signalling of both cytokines, key and central drivers of type 2 inflammation in multiple diseases.^15^ Preclinical evidence indicates that dupilumab blocks IL‐4/IL‐13‐induced B cell IgE isotype class switching and eosinophil infiltration at sites of inflammation, and expression of IL‐5, *CCL11*, *CCL17*, *CCL24*, *CCL2*, *CXCL1* and inducible nitric oxide synthase, the latter resulting in reduction in airway nitric oxide.^15^ Assessment of biomarkers of type 2 inflammation has the potential to provide an insight into the impact of therapy on the underlying disease pathogenesis and complements existing severity measures, thus aiding in identification of patients who may be more appropriate for the targeted biologic therapy within heterogenous disease populations.^16^ In previous clinical studies in asthma, dupilumab has shown greater efficacy in patients with higher levels of blood eosinophils and FeNO—biomarkers of type 2 inflammation.^17^, ^18^ We hypothesized that levels of circulating biomarkers in type 2 inflammatory diseases where IL‐4 and/or IL‐13 play critical role would be modulated by dupilumab. To investigate this, we evaluated the effect of dupilumab on type 2 inflammatory biomarkers, with the objective of demonstrating a similar suppression of type 2 inflammation across several diseases shown to respond to dupilumab therapy.

## 2 METHODS

### 2.1 Studies and patients

Data were extracted from six randomized, double‐blind, placebo‐controlled dupilumab studies. Three were phase 3 studies in adults with moderate‐to‐severe AD: 16‐week LIBERTY AD SOLO‐1 (*N* = 671, NCT02277743) and SOLO‐2 (*N* = 708, NCT02277769) studies,^19^, ^20^ and the 52‐week LIBERTY AD CHRONOS (*N* = 740, NCT02260986).^21^ The 52‐week, phase 3 LIBERTY ASTHMA QUEST study (*N* = 1902, NCT02414854)^22^ evaluated patients (aged ≥12 years) with uncontrolled, moderate‐to‐severe asthma, whereas the 52‐week, phase 3 LIBERTY NP SINUS‐52 study (*N* = 448, NCT02898454)^23^ was conducted in adults with severe CRSwNP. The 12‐week, phase 2, proof‐of‐concept (PoC) EoE study (NCT02379052)^24^ was conducted in 47 adults with active EoE.

Patients were inadequately controlled on medium‐to‐high‐dose inhaled steroids (asthma);^19^ topical steroids or topical therapy (AD);^20^, ^21^, ^22^ intranasal steroids (CRSwNP);^23^ or refractory to proton pump inhibitors (EoE).^24^ Dupilumab 300 mg every 2 weeks was administered subcutaneously in the phase 3 studies^19^, ^20^, ^21^, ^22^, ^23^ and 300 mg weekly in the phase 2 EoE study.^24^ The asthma^22^ and AD studies^19^, ^20^, ^21^ also had regimens of 200 mg every 2 weeks and 300 mg weekly, respectively; the CRSwNP study included a regimen of 300 mg every 2 weeks followed by 300 mg every 4 weeks. For the purpose of this analysis and to facilitate a fair comparison, only patients receiving 300 mg every 2 weeks are included.

All studies were conducted in accordance with the Declaration of Helsinki and the International Conference on Harmonization's good clinical practice guideline. All study documents and procedures were approved by institutional review board/ethics committees at each study site. All patients provided written informed consent before participating in the study.

Clinical data, study design, patient population details and Consolidated Standards of Reporting Trials diagrams were previously published for all studies.^19^, ^20^, ^21^, ^22^, ^23^, ^24^, ^25^

### 2.2 Assessments

Change from baseline over time in the following type 2 biomarkers was assessed and reported in the QUEST and SINUS‐52 studies as pre‐specified end‐points:^22^, ^23^ serum TARC (human TARC Quantikine ELISA kit; R&D Systems); plasma eotaxin‐3 (human eotaxin‐3 Quantikine ELISA kit; R&D Systems); serum total IgE (ImmunoCAP^®^ fluorescence enzyme immunoassay method; Phadia AB); serum periostin (ELISA kit; Shino‐Test); and blood eosinophil count (measured as part of the standard 5‐part white blood count differential cell count on a haematology autoanalyzer). QUEST also assessed FeNO (NIOX instrument; Aerocrine AB) and eosinophil cationic protein (ECP); (ImmunoCAP^®^ fluorescence enzyme immunoassay method; Phadia AB). CHRONOS, SOLO‐1 and SOLO‐2 also assessed the effect of treatment on serum TARC, serum total IgE as exploratory end‐points and blood eosinophils as pre‐specified laboratory testing.^19^, ^20^, ^21^ The EoE PoC study assessed the effect of treatment on serum TARC, serum total IgE, serum periostin and blood eosinophils from samples collected for exploratory research.^24^ Allergen‐specific IgE levels were also measured in the assessed studies. However, these data have not been included in the current analysis, as kinetics are dependent on allergen exposure (eg season, region).^20^

### 2.3 Statistical analysis

Consistent with previous analyses, biomarker levels were analysed using the observed values with censoring after rescue treatment use for CHRONOS, SOLO‐1, SOLO‐2 and PoC safety populations; observed treatment values without censoring in the SINUS‐52‐exposed population; and observed values without censoring in QUEST‐exposed population. The rationale behind censoring post–rescue treatment values in the AD and EoE studies was to remove any effect that could not be attributed to dupilumab, whereas in the asthma and CRSwNP studies, the preference was to evaluate dupilumab in a real world–like setting.

For this analysis, median absolute values, median change from baseline, median percentage change from baseline and corresponding mean values were analysed in dupilumab and matched placebo arms for all available biomarkers at each visit. Changes from baseline were summarized by descriptive statistics. Differences between dupilumab and placebo were analysed using a rank analysis of covariance model. Covariates included the corresponding baseline value and the stratification variables used in each of the studies. Given the distribution of biomarkers, mean changes from baseline were reported as descriptive statistics, without *p‐values*. A *p*‐value of less than .05 for the comparison between dupilumab and placebo (within each study) was considered statistically significant.

## 3 RESULTS

### 3.1 Patients

Appendix S1 summarizes baseline demographics and comorbid type 2 inflammatory diseases in all studies. Individual study treatment groups had similar baseline demographics and disease characteristics. Type 2/Th2 immune‐mediated comorbidities were frequent, particularly allergic rhinitis (AR; 42.5%–70.1%) in patients across all indications, and asthma (33.3%–59.5%) in AD and CRSwNP patients. Most EoE patients had comorbid AR or food allergy. At baseline, higher median concentrations of serum TARC (1936.5–4030.1 pg/mL) and total IgE (2396.0–4925.5 IU/mL) were observed in AD patients compared with asthma, CRSwNP and EoE patients (293.0–333.5 pg/mL [TARC]; 67.0–178.5 IU/mL [IgE]) (Table 1). Median baseline levels of eosinophils were also relatively higher in AD (0.40–0.45 Giga/L) than in asthma (0.25–0.27 Giga/L), CRSwNP (0.34–0.36 Giga/L) or EoE (0.30–0.40 Giga/L) (Table 1). Median levels of eotaxin‐3 were only assessed in CRSwNP and asthma patients, with numerically higher baseline concentrations observed in CRSwNP (60.50–61.55 and 37.45–38.30 pg/mL, respectively). Periostin (not measured in the AD studies presented here) ranged from 56.20 to 56.95 ng/mL in EoE patients to 69.70 to 71.00 ng/mL and 99.60 to 104.00 ng/mL in asthma and CRSwNP patients, respectively (Table 1).

**TABLE 1:** Concentrations of circulating type 2 inflammatory biomarkers at baseline and effect of dupilumab on concentrations at the end of treatment for patients with AD, asthma, CRSwNP and EoE

Type 2 disease	Atopic dermatitis		Asthma		Chronic rhinosinusitis with nasal polyps		Atopic dermatitis		Eosinophilic esophagitis	
Study name	CHRONOS		QUEST		SINUS‐52		SOLO‐1 and SOLO‐2		EoE PoC	
Study duration	52 weeks		52 weeks		52 weeks		16 weeks		12 weeks	
Treatment group	Placebo qw + TCS	Dupilumab 300 mg q2w + TCS	Placebo 2 ml q2w + ICS/LABA	Dupilumab 300 mg q2w + ICS/LABA	Placebo q2w + INCS	Dupilumab 300 mg q2w + INCS	Placebo qw	Dupilumab 300 mg q2w	Placebo qw	Dupilumab 300 mg qw
**Serum TARC (pg/ml)**										
*n*	313	110	319	622	149	147	454	464	24	22
Baseline median value (95% CI)	2868.20 (2099.10 to 3665.10)	4030.10 (2989.00 to 5137.10)	300 (277.00 to 334.00)	295 (277.00 to 315.00)	304.00 (267.00 to 341.00)	303.00 (275.00 to 347.00)	2195.00 (1840.00 to 2636.00)	1936.50 (1637.00 to 2573.00)	333.50 (225.00 to 396.00)	293.00 (196.00 to 377.00)
*n*	120	84	248	479	127	139	194	361	19	22
Median value (95% CI) at end of treatment	965.10 (729.10 to 1227.80)	359.95 (313.40 to 454.50)	303.50 (281.00 to 337.00)	201.00 (189.00 to 214.00)	246.00 (227.00 to 296.00)	187.00 (165.00 to 210.00)	1025.00 (755.00 to 1231.00)	385.00 (355.00 to 417.00)	292.00 (196.00 to 398.00)	197.50 (133.00 to 292.00)
*n*	119	84	246	473	126	137	193	360	19	21
Median change from baseline (95% CI) at end of treatment	−987.40 (−1218.00 to −552.20)	−4238.55 (−6103.50 to −2795.60)	8.5 (−13.00 to 19.00)	−85.00 (−95.00 to −73.00)	−9.50 (−26.00 to 2.00)	−111.00 (−130.00 to −94.00)	−108.00 (−257.00 to −40.00)	−1297.00 (−1602.00 to −1002.00)	−13.00 (−51.00 to 28.00)	−90.00 (−111.00 to −15.00)
*p*‐value dupilumab vs placebo		<.0001		<.0001		<.0001		<.0001		.0143
*n*	119	84	246	473	126	137	193	360	19	21
Median % change from baseline (95% CI) at end of treatment	−53.6 (−61.7 to −43.8)	−88.6 (−93.6 to −82.0)	2.6 (−4.03 to 7.11)	−33.51 (−36.00 to −29.84)	−2.75 (−8.02 to 0.85)	−39.44 (−43.17 to −35.08)	−16.4 (−26.0 to −6.2)	−78.9 (−81.5 to −76.6)	−8.2 (−12.8 to 13.5)	−24.8 (−38.7 to −11.7)
*p*‐value dupilumab vs placebo		<.0001		<.0001		<.0001		<.0001		.006
**Plasma eotaxin‐3 (pg/ml)**										
*n*			318	625	150	146				
Baseline median value (95% CI)			37.45 (34.60 to 40.10)	38.30 (36.00 to 40.10)	61.55 (51.80 to 71.00)	60.50 (55.10 to 72.40)				
*n*			246	473	127	139				
Median (95% CI) at end of treatment			38.45 (35.50 to 42.60)	23.50 (22.40 to 25.70)	62.90 (53.70 to 68.80)	28.5 (25.10 to 31.20)				
*n*			243	469	127	136				
Median change from baseline (95% CI) at end of treatment			−0.10 (−1.70 to 2.00)	−12.90 (−15.80 to −11.40)	3.6 (−0.70 to 9.10)	−26.70 (−33.30 to −21.50)				
*p*‐value dupilumab vs placebo				<.0001		<.0001				
*n*			243	469	127	136				
Median % change from baseline (95% CI) at end of treatment			−0.16 (−4.85 to 8.97)	−38.24 (−42.42 to −33.70)	8.3 (−1.75 to 19.64)	−51.47 (−57.21 to −45.24)				
*p*‐value dupilumab vs placebo				<.0001		<.0001				
**Serum total IgE (IU/ml)**										
*n*	312	110	318	626	150	148	456	465	24	23
Baseline median (95% CI)	3477.00 (2471.00 to 4757.00)	4925.50 (3038.00 to 10 000.00)	178.50 (138.00 to 213.00)	174.00 (152.00 to 200.00)	139.00 (104.00 to 172.00)	122.50 (98.00 to 157.00)	2849.00 (2174.00 to 3817.00)	2396.00 (2001.00 to 3025.00)	126.50 (58.00 to 254.00)	67 (42.00 to 220.00)
*n*	117	83	251	482	128	140	196	366	19	23
Median (95% CI) at end of treatment	2257 (1327.00 to 3711.00)	951.00 (520.00 to 1648.00)	176.00 (136.00 to 217.00)	48.5 (40.00 to 55.00)	144.00 (115.00 to 191.00)	29.5 (23.00 to 40.00)	1592.00 (927.00 to 3022.00)	1107.00 (739.00 to 1518.00)	159.00 (64.00 to 388.00)	53 (33.00 to 147.00)
*n*	116	83	248	478	128	139	196	366	19	23
Median change from baseline (95% CI) at end of treatment	0 (−7.00 to 0)	−2549.00 (−5481.00 to −735.00)	−2.50 (−7.00 to 1.00)	−116.50 (−135.00 to −102.00)	4 (0 to 8.00)	−84.00 (−106.00 to −62.00)	17.10 (3.00 to 75.00)	−976.00 (−1331.00 to −696.00)	9.00 (−4.00 to 28.00)	−17.00 (−40.00 to −9.00)
*p*‐value dupilumab vs placebo		<.0001		<.0001		<.0001		<.0001		<.0001
*n*	116	83	248	478	128	139	196	366	19	23
Median % change from baseline (95% CI) at end of treatment	0 (−6.5 to 0)	−76.7 (−80.9 to −72.7)	−4.42 (−10.00 to 0.51)	−69.97 (−72.22 to −67.60)	7.54 (0 to 14.57)	−71.43 (−75.61 to −67.39)	7.8 (2.1 to 15.1)	−46.5 (−48.4 to −44.4)	8.3 (−6.9 to 18.0)	−24.8 (−33.2 to −14.5)
*p*‐value dupilumab vs placebo		<.0001		<.0001		<.0001		<.0001		<.0001
**Serum periostin (ng/ml)**										
*n*			319	629	150	147			24	22
Baseline median (95% CI)			71.00 (66.30 to 75.10)	69.70 (67.30 to 72.80)	104.00 (95.70 to 113.00)	99.60 (86.60 to 114.00)			56.95 (49.70 to 68.20)	56.20 (48.70 to 76.90)
*n*			253	490	120	128			18	23
Median (95% CI) at end of treatment			66.50 (61.20 to 72.20)	55.5 (53.70 to 57.60)	94.15 (80.80 to 104.00)	61.55 (57.60 to 65.60)			61.60 (53.90 to 86.80)	47.00 (44.20 to 55.90)
*n*			251	488	120	127			18	22
Median change from baseline (95% CI) at end of treatment			−4.10 (−6.40 to −1.80)	−12.45 (−14.30 to −10.40)	−3.15 (−8.50 to 1.30)	−39.00 (−52.70 to −30.40)			7.5 (−3.70 to 14.10)	−8.95 (−13.30 to 2.10)
*p*‐value dupilumab vs placebo				<.0001		<.0001				.0016
*n*			251	488	120	127			18	22
Median % change from baseline (95% CI) at end of treatment			−6.94 (−9.93 to −3.16)	−18.93 (−21.48 to −16.23)	−3.52 (−8.29 to 1.62)	−41.08 (−46.22 to −31.14)			10.1 (−6.5 to 25.8)	−13.6 (−22.5 to 3.0)
*p*‐value dupilumab vs placebo				<.0001		<.0001				.0005
**Blood eosinophils (Giga/L)**										
*n*	314	110	320	632	150	148	454	465	24	23
Baseline median (95% CI)	0.42 (0.36 to 0.49)	0.45 (0.37 to 0.52)	0.27 (0.24 to 0.30)	0.25 (0.22 to 0.28)	0.36 (0.32 to 0.40)	0.34 (0.30 to 0.40)	0.40 (0.40 to 0.50)	0.40 (0.40 to 0.50)	0.40 (0.20 to 0.50)	0.30 (0.20 to 0.40)
*n*	120	83	245	476	129	140	195	360	21	22
Median (95% CI) at end of treatment	0.28 (0.25 to 0.32)	0.3 (0.23 to 0.35)	0.25 (0.22 to 0.29)	0.19 (0.17 to 0.22)	0.34 (0.29 to 0.39)	0.26 (0.21 to 0.31)	0.3 (0.30 to 0.40)	0.3 (0.30 to 0.40)	0.3 (0.20 to 0.60)	0.15 (0.10 to 0.20)
*n*	119	83	245	476	129	139	194	360	21	22
Median change from baseline (95% CI) at end of treatment	−0.08 (−0.16 to −0.04)	−0.15 (−0.20 to −0.09)	−0.03 (−0.04 to −0.01)	−0.03 (−0.04 to −0.01)	−0.03 (−0.07 to 0.02)	−0.07 (−0.11 to −0.04)	−0.10 (−0.10 to 0)	0 (−0.10 to 0)	0 (−0.20 to 0.10)	−0.10 (−0.20 to −0.10)
*p*‐value dupilumab vs placebo		.5718		.2916		.0814		.0755		.0455
*n*	119	83	242	469	128	139	190	346	20	21
Median % change from baseline (95% CI) at end of treatment	−29.8 (−36.8 to −18.2)	−40.7 (−50.0 to −31.5)	−11.44 (−17.78 to −3.57)	−14.58 (−20.00 to −7.69)	−11.88 (−17.86 to 5.88)	−29.41 (−40.00 to −16.33)	−20.0 (−30.8 to 0)	0 (−15.8 to 0)	−12.5 (−50.0 to 33.3)	−50.0 (−50.0 to −33.3)
*p*‐value dupilumab vs placebo		.1817		.0552		.0174		.0552		.0282

Note

Grey cells indicate that data were not collected or available for these patients.

Abbreviations: AD, atopic dermatitis; CI, confidence interval; CRSwNP, chronic rhinosinusitis with nasal polyps; EoE, eosinophilic esophagitis; ICS, inhaled corticosteroids; INCS, intranasal corticosteroids; IU, international units; LABA, long‐acting β_2_‐agonist; PoC, proof of concept; q2w, every 2 weeks; qw, weekly; TARC, thymus and activation‐regulated chemokine; TCS, topical corticosteroids.

### 3.2 Effect of dupilumab on type 2 inflammatory biomarkers

Compared with placebo, dupilumab significantly suppressed circulating levels of most type 2 inflammatory biomarkers across all studies/disease types where a particular biomarker was assessed (Table 1). Specifically, dupilumab versus placebo significantly suppressed serum TARC and total IgE levels in patients with asthma, AD, CRSwNP and EoE. Plasma eotaxin‐3 and serum periostin were significantly suppressed in asthma and CRSwNP; periostin was also significantly suppressed in EoE patients. Blood eosinophil responses to dupilumab varied by disease, with minimal changes observed in median values in AD, transient increases followed by decreases to below‐baseline levels in asthma and CRSwNP, and significant suppression observed in EoE. Mean absolute and percentage changes generally followed a similar course to median values, although in AD, transient mean increases in eosinophils from baseline were observed (Appendix S2).

### 3.3 Serum TARC

**FIGURE 2:**
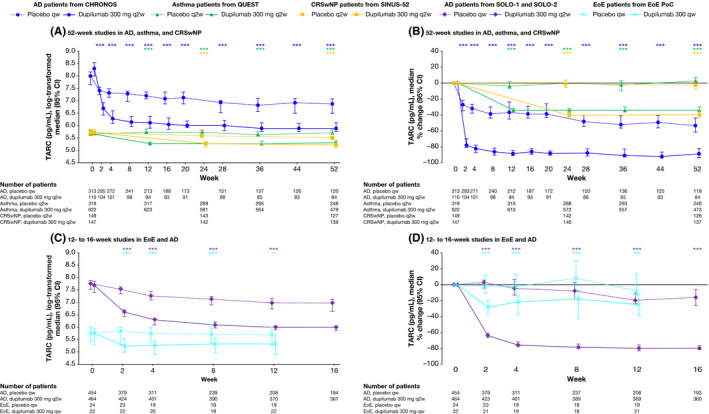
The effect of dupilumab on concentrations of serum TARC (pg/mL). *p < .05; **p < .01, ***p < .001 vs matched placebo. *p*‐values represent differences between dupilumab and placebo in change from baseline (A and C) or percentage change from baseline (B and D) and were analysed using a rank ANCOVA model. CHRONOS, SOLO‐1 and SOLO‐2 included the corresponding baseline values as covariates and the treatment, geographic region, baseline Investigator Global Assessment (IGA) strata and study identifier as fixed factors. EoE PoC included the corresponding baseline values as covariates and the treatment, baseline Straumann Dysphagia Instrument (SDI) strata and study identifier as fixed factors. QUEST included the corresponding baseline value, age, sex, geographic region (pooled country), baseline eosinophil strata and baseline inhaled corticosteroid dose level as covariates. SINUS‐52 included the corresponding baseline value, age, geographic region, asthma/nonsteroidal anti‐inflammatory drugs–exacerbated respiratory disease (NSAID‐ERD) status and prior surgery history as covariates. AD, atopic dermatitis; ANCOVA, analysis of covariance; CI, confidence interval; CRSwNP, chronic rhinosinusitis with nasal polyps; EoE, eosinophilic esophagitis; PoC, proof of concept; qw, weekly; q2w, every 2 weeks; TARC, thymus and activation‐regulated chemokine

Treatment with dupilumab was associated with sustained reductions in median serum levels of TARC (Table 1 and Figure 2) in all four diseases. In AD patients, rapid, significant reductions versus placebo were evident from week 2, with maximum effects observed at week 8, and maintained until the last assessment at week 52 or 16. Significant reductions, sustained throughout the treatment period, were also observed by the first assessment in patients with EoE (at week 2) and in patients with asthma and CRSwNP (weeks 12 and 24, respectively). At the end of the 52‐week treatment period in the AD, asthma and CRSwNP studies, median percentage changes from baseline in serum TARC levels were between −88.6% and −33.5% for dupilumab groups, compared with −53.6% and 2.6% for placebo groups (all *p* < .0001). In the pooled SOLO studies at week 16, the median percentage change in TARC was −78.9% in the dupilumab group versus −16.4% in the placebo group (*p* < .0001), and at the end of the EoE study at week 12, −24.8% in the dupilumab group versus −8.2% in the placebo group (*p* = .006).

### 3.4 Plasma eotaxin‐3

**FIGURE 3:**
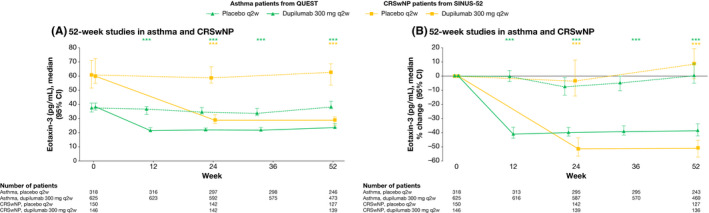
The effect of dupilumab on concentrations of plasma eotaxin‐3 (pg/mL). ***p < .001 vs matched placebo. *p*‐values represent differences between dupilumab and placebo in change from baseline (A) or percentage change from baseline (B) and were analysed using a rank ANCOVA model. QUEST included the corresponding baseline value, age, sex, geographic region (pooled country), baseline eosinophil strata and baseline inhaled corticosteroid dose level as covariates. SINUS‐52 included the corresponding baseline value, age, geographic region (pooled country), asthma/NSAID‐ERD status and prior surgery history as covariates. ANCOVA, analysis of covariance; CI, confidence interval; CRSwNP, chronic rhinosinusitis with nasal polyps; NSAID‐ERD, nonsteroidal anti‐inflammatory drugs–exacerbated respiratory disease; q2w, every 2 weeks

In asthma and CRSwNP patients, dupilumab significantly reduced plasma eotaxin‐3 versus placebo by the first assessment (weeks 12 and 24, respectively; both *p* < .0001) (Table 1 and Figure 3). This reduction was sustained to the end of treatment in both studies: at week 52, the median percentage change from baseline in plasma eotaxin‐3 in dupilumab‐ versus placebo‐treated patients was −38.24% versus −0.16% in asthma patients and −51.47% versus 8.30% in CRSwNP patients (both *p* < .0001 versus placebo). Eotaxin‐3 was not assessed in the AD or EoE studies.

### 3.5 Serum total IgE

**FIGURE 4:**
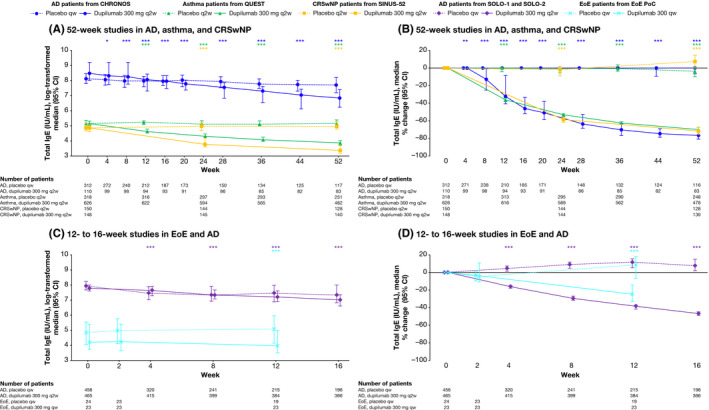
The effect of dupilumab on concentrations of serum total IgE (IU/mL). *p < .05; **p < .01, ***p < .001 vs matched placebo. *P*‐values represent differences between dupilumab and placebo in change from baseline (A and C) or percentage change from baseline (B and D) and were analysed using a rank ANCOVA model. CHRONOS, SOLO‐1 and SOLO‐2 included the corresponding baseline values as covariates and the treatment, geographic region, baseline IGA strata and study identifier as fixed factors. EoE PoC included the corresponding baseline values as covariates and the treatment, baseline SDI strata and study identifier as fixed factors. QUEST included the corresponding baseline value, age, sex, geographic region (pooled country), baseline eosinophil strata and baseline inhaled corticosteroid dose level as covariates. SINUS‐52 included the corresponding baseline value, age, geographic region (pooled country), asthma/NSAID‐ERD status and prior surgery history as covariates. AD, atopic dermatitis; CI, confidence interval; CRSwNP, chronic rhinosinusitis with nasal polyps; EoE, eosinophilic esophagitis; NSAID‐ERD, nonsteroidal anti‐inflammatory drugs–exacerbated respiratory disease; PoC, proof of concept; qw, weekly; q2w, every 2 weeks; SDI, Straumann Dysphagia Instrument

Dupilumab versus placebo significantly reduced concentrations of serum total IgE in AD, asthma, CRSwNP and EoE during treatment; however, across all type 2 diseases, the effect was more gradual than with the other type 2 biomarkers (Table 1 and Figure 4). In the 52‐week studies, maximum reductions in median percentage change in serum total IgE from −70.0% to −76.7% were observed in AD (CHRONOS), asthma and CRSwNP patients in the dupilumab‐treated groups by the end of treatment (all *p* < .0001 versus placebo). In the shorter studies, maximum reductions of −46.5% were observed in AD patients (SOLO‐1 & SOLO‐2) at week 16 and −24.8% in EoE patients at week 12 (both *p* < .0001 versus placebo). Serum total IgE levels in the placebo groups remained relatively constant during treatment across all studies.

### 3.6 Serum periostin

**FIGURE 5:**
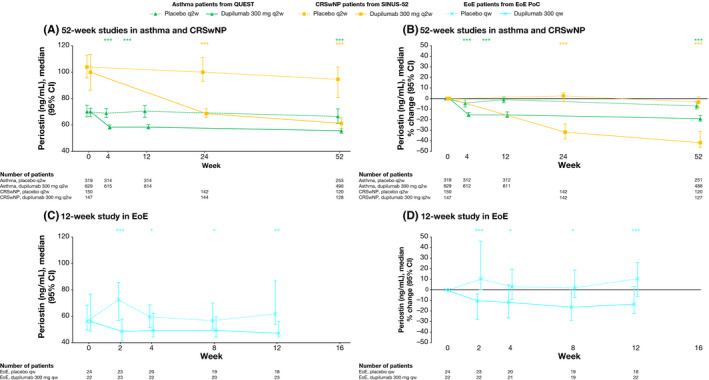
The effect of dupilumab on concentrations of serum periostin (ng/mL). *p < .05; **p < .01; ***p < .001 vs matched placebo. *p*‐values represent differences between dupilumab and placebo in change from baseline (A and C) or percentage change from baseline (B and D) and were analysed using a rank ANCOVA model. EoE PoC included the corresponding baseline values as covariates and the treatment, baseline SDI strata and study identifier as fixed factors. QUEST included the corresponding baseline value, age, sex, geographic region (pooled country), baseline eosinophil strata and baseline inhaled corticosteroid dose level as covariates. SINUS‐52 included the corresponding baseline value, age, geographic region (pooled country), asthma/NSAID‐ERD status and prior surgery history as covariates. ANCOVA, analysis of covariance; CI, confidence interval; CRSwNP, chronic rhinosinusitis with nasal polyps; EoE, eosinophilic esophagitis; NSAID‐ERD, nonsteroidal anti‐inflammatory drugs–exacerbated respiratory disease; PoC, proof of concept; qw, weekly; q2w, every 2 weeks; SDI, Straumann Dysphagia Instrument

In patients with asthma, CRSwNP and EoE, dupilumab treatment was associated with rapid, sustained reductions in serum levels of periostin versus placebo. Significant reductions versus placebo were evident from the earliest assessment (week 2 or 4), with the maximum effect observed at week 52 (Table 1 and Figure 5). At week 52, median percentage changes from baseline in periostin in asthma and CRSwNP patients were −18.93% and −41.08% for dupilumab groups, versus −6.94% and −3.52% for placebo groups (both *p* < .0001 versus placebo). In EoE patients, median percentage change in periostin by week 12 was −13.6% with dupilumab versus 10.1% with placebo (*p* = .0005).

### 3.7 Blood eosinophils

**FIGURE 6:**
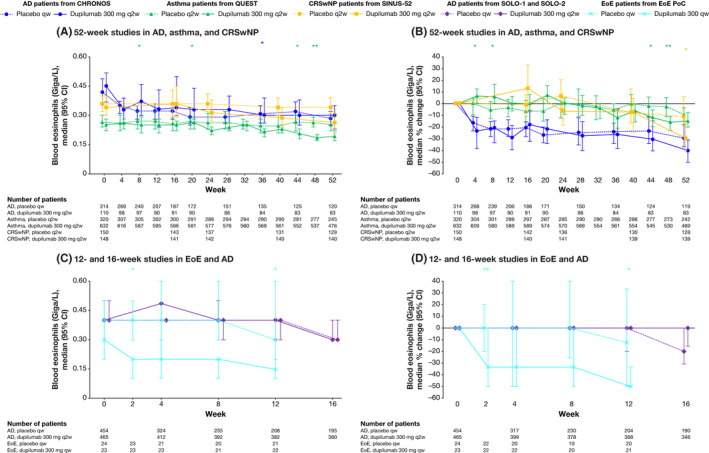
The effect of dupilumab on concentrations of blood eosinophils (Giga/L). *p < .05; **p < .01; ***p < .001 vs matched placebo. *p*‐values represent differences between dupilumab and placebo in change from baseline (A and C) or percentage change from baseline (B and D) and were analysed using a rank ANCOVA model. CHRONOS, SOLO‐1 and SOLO‐2 included the corresponding baseline values as covariates and the treatment, geographic region, baseline IGA strata and study identifier as fixed factors. EoE PoC included the corresponding baseline values as covariates and the treatment, baseline SDI strata and study identifier as fixed factors. QUEST included the corresponding baseline value, age, sex, geographic region (pooled country), baseline eosinophil strata and baseline inhaled corticosteroid dose level as covariates. SINUS‐52 included the corresponding baseline value, age, geographic region (pooled country), asthma/NSAID‐ERD status and prior surgery history as covariates. AD, atopic dermatitis; ANCOVA, analysis of covariance; CI, confidence interval; CRSwNP, chronic rhinosinusitis with nasal polyps; EoE, eosinophilic esophagitis; NSAID‐ERD, nonsteroidal anti‐inflammatory drugs–exacerbated respiratory disease; PoC, proof of concept; qw, weekly; q2w, every 2 weeks; SDI, Straumann Dysphagia Instrument

In dupilumab‐treated patients with asthma and CRSwNP, transient elevations in blood eosinophils were observed in a subset of patients (median percentage change from baseline), followed by a decrease to near‐baseline levels by the end of treatment (Table 1 and Figure 6A,B), with mean eosinophil values following a similar course (Appendix S2 and S3). In patients with EoE, dupilumab reduced blood eosinophil counts at all time‐points, with a maximum reduction at week 12 of −50.0% compared with −12.5% in the placebo‐treated group (*p* = .0282) (Table 1 and Figure 6C,D). In the 16‐week AD studies, the effect of dupilumab treatment on blood eosinophils did not differ significantly from that of placebo at any time‐point despite a transient increase in median blood eosinophil levels at week 4 (Figure 6C,D). In the 52‐week AD study, median absolute values of eosinophils initially fell from baseline to week 4 in both dupilumab‐ and placebo‐treated groups, then fluctuated thereafter but remained below baseline levels; no statistically significant differences were observed between treatment groups at any time‐point (Figure 6A,B). The corresponding mean eosinophil levels also fell from baseline to week 4, before rising to slightly above baseline at week 8, falling below baseline at week 12, and generally declining thereafter (Appendix S2 and S3).

## 4 DISCUSSION

Atopic and allergic diseases share common underlying type 2 inflammatory mechanisms manifesting as disease in diverse organs. This analysis evaluated whether the effects of dupilumab on systemic type 2 inflammatory biomarkers were analogous in patients with AD (SOLO‐1 and SOLO‐2, 16 weeks; and CHRONOS, 52 weeks), asthma (QUEST, 52 weeks), CRSwNP (SINUS‐52, 52 weeks) and EoE (PoC, 12 weeks).

The type 2 inflammatory biomarkers assessed (serum TARC, plasma eotaxin‐3, serum total IgE, serum periostin and peripheral blood eosinophils) reflect a range of processes involved in type 2 inflammation, though their baseline levels vary according to the underlying type 2 disease. The reductions observed in most circulating biomarkers with dupilumab treatment were consistent across indications. Consistent with the mechanism of action of dupilumab,^15^ these findings demonstrate the key and central roles of IL‐4 and IL‐13 in type 2 inflammation and further support the hypothesis that dual blockade of these cytokine pathways has important anti‐inflammatory effects in multiple type 2 inflammatory diseases. However, while IL‐4 and IL‐13 play central roles, varying effects of these cytokines resulted in differences in the effector cells involved in the pathogenesis of each disease, as evidenced by differences in biomarkers outlined below.

All of the patient populations included in these studies had moderate‐to‐severe disease. As previously reported,^26^ AD is characterized by higher baseline levels of type 2 biomarkers (eg TARC and total IgE) than asthma, CRSwNP and EoE. This may be related to the more severe barrier dysfunction that occurs in AD, with open skin lesions allowing deeper allergen penetration and presentation to submucosal lymphoid elements. In AD, TARC is primarily secreted from keratinocytes, and serum TARC levels are known to be correlated with the severity of disease,^27^ although not predictive of clinical response to dupilumab.^28^ In the airways, TARC is secreted by respiratory epithelial cells, and serum concentrations in patients with asthma and CRSwNP, while overall elevated, substantially overlap with concentrations in healthy subjects^29^ and do not correlate with changes in forced expiratory volume in one second (FEV_1_) during treatment with dupilumab.^17^ Across diseases, dupilumab treatment resulted in reductions in TARC, eotaxin‐3 and periostin concentrations, with maximum effect achieved as early as first assessment and suppression maintained throughout treatment. This was particularly evident for TARC concentrations in AD patients, which reflects these patients’ markedly higher baseline levels. Eosinophil chemotaxis is regulated by the local production of eotaxins. Although circulating eotaxin‐3 was not measured in the AD or EoE studies of this analysis, suppression of eotaxin‐3 messenger RNA was observed in the skin of AD patients and oesophageal biopsies of EoE patients treated with dupilumab in other studies.^30^, ^31^, ^32^ Similarly, significant suppression of periostin has been observed in dupilumab‐treated AD patients though it was not assessed here.^30^ Further, previous phase 2 asthma and CRSwNP trials with earlier sampling showed marked decreases in TARC and eotaxin‐3 by week 2 of treatment.^33^, ^34^ These data collectively confirm that up‐regulation of TARC, eotaxin‐3 and periostin during type 2 inflammation is substantially dependent on IL‐4 and IL‐13 signalling, consistent with known regulation of their expression by IL‐4R‐mediated activation of the gene transcription factor STAT6.^35^, ^36^, ^37^

In contrast, reductions in serum total IgE progressed more gradually in all disease settings, with greater effects observed during longer treatment durations. In AD, for example, the exact role of IgE in pathogenesis remains unclear, although adults with AD have a higher incidence of serum IgE autoantibodies that are autoreactive to proteins in skin. In principle, mast cells and other cells with IgE receptors (eg eosinophils) in the skin can be sensitized and degranulate in response to allergens entering the skin either due to an intrinsic barrier defect or excoriation. Furthermore, expression of type I IgE receptor (FcεRI) is by Langerhans cells, and inflammatory dendritic epidermal cells are up‐regulated by IgE. Through receptor‐bound IgE molecules specific for certain allergens, these cells can uptake and process allergens, and present the processed allergens to T cells, resulting in local T cell activation.^38^ Similarly, in asthma, IgE‐FcεRI binding in the bronchial mucosa results in mast cell activation and degranulation, driving a hypersensitivity reaction in the airways, including smooth muscle contraction, oedema and hypersecretion of mucus.^39^ Dupilumab has the potential to modulate IgE‐related mechanisms by diminishing infiltrates of cells expressing FcεRI,^30^ including mast cells, eosinophils and Langerhans cells. Suppression of mast cell infiltration and activation may also be one of the mechanisms by which dupilumab diminishes itch in patients with AD for example, as dupilumab treatment has been associated with reduced monocyte chemotactic protein‐4, which is substantially up‐regulated in AD skin.^30^ The slower effect of dupilumab on IgE production compared with the other type 2 biomarkers suggests that IL‐4 and IL‐13 blockade of immunoglobin class switching in B cells may not immediately affect plasmablasts and long‐lived plasma cells pre‐programmed to produce IgE. Thus, a gradual decline in serum IgE progressing as much as a year after initiating dupilumab treatment may be a function of both diminished production of new IgE‐secreting plasmablasts as well as gradual turnover of pre‐existing IgE‐secreting cells.^40^, ^41^

Blood eosinophil responses to dupilumab varied by disease type. Transient increases in a subset of patients were followed by decreases to below‐baseline levels in asthma and CRSwNP. As previously published for QUEST, transient increases were also observed in levels of ECP, a ribonuclease released during eosinophil degranulation.^22^ No meaningful change was observed in AD patients treated with concomitant topical corticosteroids, but transient increases were seen in the monotherapy studies. In contrast, significant decreases in circulating eosinophils were observed in dupilumab‐treated EoE patients, although this finding needs to be corroborated in a larger sample of patients. Based on preclinical work, the transient increase in eosinophils observed in asthma and CRSwNP patients may be related to blockade of IL‐4Rα with dupilumab reducing eotaxin production, thus inhibiting eosinophil chemotaxis and tissue infiltration.^15^ This hypothesis is further supported by the suppression of eotaxin‐3 messenger RNA expression observed in AD lesional skin^30^, ^31^ and EoE oesophageal biopsies^31^ following dupilumab treatment. In contrast to anti–IL‐5 therapeutic antibodies, dupilumab does not appear to have direct effects on bone marrow or circulating eosinophil counts,^5^, ^42^ but it does lead to a marked decrease of eosinophils in tissue by different mechanisms, including eotaxin and *vascular cell adhesion molecule 1* (VCAM1) down‐regulation.^15^, ^22^, ^24^ The trafficking of eosinophils into tissues is dependent on the adhesion protein VCAM1,^43^ the expression of which is regulated by STAT6 and IL‐4. The transient increase in blood eosinophils observed in some patients with asthma and CRSwNP during dupilumab treatment^22^, ^23^ tends to occur in patients with higher baseline eosinophil counts, which may suggest these individuals have higher baseline eosinophil production rates. If this is the case, blocking eosinophil recruitment into tissue, for example. by reduced expression of eotaxin‐3 or VCAM1 by endothelial cells,^44^ without affecting eosinophil production may explain the transient increase in peripheral blood eosinophil counts observed in a subset of patients in some indications. It should be noted that most increases were not associated with clinical symptoms.

There is increasing evidence that nitric oxide plays a key role in modulating type 2 inflammation and in regulating type 2 immune responses, as an endogenous modulator of airway function and as a proinflammatory and immunomodulatory mediator.^11^, ^45^ In the context of asthma, the inflammatory response results in increased symptoms and airway obstruction.^11^, ^46^ Increased levels of exhaled nitric oxide in asthma are often associated with airway eosinophilic inflammation and may also be associated with exacerbations and disease severity.^11^ As previously published, dupilumab was associated with greater reductions in FeNO levels over the course of the treatment period in QUEST, compared with placebo.^22^ Further, we have also previously reported a significant correlation between suppression of FeNO and improvement in FEV_1_ in dupilumab asthma trials.^47^

The data on type 2 inflammation suppression and efficacy of dupilumab observed in the indications tested to date demonstrate that the common underlying pathological mechanism in these diseases is mediated by IL‐4 and IL‐13, and that suppression of inflammatory mediators by dual blockade of these cytokines and their downstream pathways consequently reduces disease activity. Significant improvements have been observed in lung function and exacerbation rates in asthma;^22^, ^42^ signs and symptoms of AD;^19^, ^20^, ^21^ sinonasal outcomes in CRSwNP;^23^ and dysphagia, oesophageal eosinophil counts and other histopathologic features in EoE.^24^ Type 2 inflammatory diseases often coexist in the same patient,^2^, ^48^, ^49^, ^50^, ^51^ and several studies have shown that dupilumab can significantly improve outcome measures in patients with comorbid type 2 diseases.^52^, ^53^, ^54^ These improvements have been associated with consistent suppression of type 2 biomarkers across type 2 inflammatory diseases.^52^, ^55^ Type 2 inflammatory diseases are defined not simply by their anatomic location but also by their common underlying pathogenesis, which is effectively the ‘treatable trait’ against which treatment can be targeted. A treatment that is effective in multiple type 2 inflammatory diseases would therefore be expected to offer important benefits to patients with several comorbidities.

One of the limitations of the current analysis was that only blood biomarkers were included. Peripheral blood levels may not be as representative of physiology within the specific target tissue and therefore, of changes in type 2 inflammation within the affected tissues underlying the disease. Analysis of biomarkers in tissue gives greater insights into local effects, including target tissues and effects on clinical disease. More invasive tissue sampling in other studies has shown that dupilumab can suppress type 2 biomarker levels in target organs, such as suppression of ECP, eotaxin‐1, eotaxin‐2, eotaxin‐3, pulmonary and activation‐regulated chemokine (PARC), IgE and IL‐13 in nasal polyp tissue of CRSwNP patients in a phase 2 study;^7^ and suppression of ECP, total IgE, eotaxin‐3 and IL‐5 in nasal secretions in SINUS‐52.^23^ Decreased eosinophil infiltration in the oesophageal tissue has also been observed in EoE patients treated with dupilumab.^24^ Similarly, skin biopsies in AD patients have shown marked local reductions in expression of type 2 inflammatory pathway genes, including the STAT6‐regulated genes eotaxin‐3, TARC and PARC (CCL‐18).^30^, ^31^

Caution should be taken when comparing the absolute values of biomarkers measured in our clinical trials versus those reported in other clinical studies. Of the various biomarkers, only the assay for serum total IgE is well standardized and subject to proficiency testing across clinical laboratories. Hence, it is preferable when making cross‐study comparisons to reference percentage changes from baseline (relative to placebo as appropriate). In particular, the assay results for the periostin ELISA used in our studies are highly correlated with the assay developed for the ELECSYS platform but systematically report higher values.^56^

Control of type 2 inflammation, as evidenced by reduced biomarkers observed in this study together with the observed clinical efficacy of dupilumab across the diseases studied and additional transcriptome analysis,^25^, ^57^ indicates that dupilumab is acting on the underlying disease pathogeneses and by reducing inflammation. The reduction in the type 2 mediators measured and described in this study also contribute to the improvement of barrier mucosal and epithelial function to a healthier phenotype, including direct effects suppressing epithelial or epidermal hyperproliferation seen in these diseases.^30^, ^32^ For example, reducing IgE in patients with asthma may restore the antiviral functions of plasmacytoid dendritic cells, thus reducing the risk of exacerbations.^3^ By disrupting the inflammatory cycle, barrier function can be restored, reducing the exposure to causative agents and thus further reducing inflammation and release of pro‐inflammatory cytokines.^58^ The consistent modulation of type 2 biomarkers with dupilumab treatment seen in these studies highlights the shared aetiology of type 2 inflammation in these diseases driven by IL‐4 and IL‐13.

## 5 CONCLUSIONS

Dupilumab consistently and significantly reduced most type 2 inflammatory biomarkers in patients with AD, asthma, CRSwNP and EoE. The data support the hypothesis that IL‐4 and IL‐13 are key and central drivers of type 2 inflammation across numerous type 2 inflammatory diseases. Added to other clinical and preclinical evidence, the findings demonstrate dupilumab inhibits a common pathophysiological mechanism fundamental to type 2 inflammatory diseases.

## CONFLICTS OF INTEREST

JDH, SH, ZC, NA, MA, AR, BS and MR are employees and shareholders of Regeneron Pharmaceuticals, Inc. NMHG is a prior employee and shareholder of Regeneron Pharmaceuticals, Inc. WB, MSR, NP and LM are employees and may hold stock and/or stock options in Sanofi. BG and GP are prior employees and may hold stock and/or stock options in Sanofi.

## AUTHOR CONTRIBUTION

JDH, SH, BS, WB, NA, MA, AR, BS, MR, NP, GP, LM and NMHG contributed to study design and data analysis or interpretation (ICMJE Criterion #1), drafting and providing critical feedback on the publication (ICMJE Criterion #2), gave final approval for submission (ICMJE Criterion #3) and agreed to be accountable for the accuracy and integrity of this work (ICMJE Criterion #4). ZC and MSR provided statistical support and contributed to data analysis or interpretation (#1), drafting and providing critical feedback on the publication (#2), gave final approval for submission (#3) and agreed to be accountable for the accuracy and integrity of this work (#4).

## ETHICAL STATEMENT

All studies were conducted in accordance with the Declaration of Helsinki and the International Conference on Harmonization's good clinical practice guideline. All study documents and procedures were approved by institutional review board/ethics committees at each study site. All patients provided written informed consent before participating in the study.

## Supporting information

Supplementary Material

## Data Availability

Qualified researchers may request access to patient level data and related study documents including the clinical study report, study protocol with any amendments, blank case report form, statistical analysis plan and dataset specifications. Patient level data will be anonymized, and study documents will be redacted to protect the privacy of our trial participants. Further details on Sanofi's data sharing criteria, eligible studies and process for requesting access can be found at: http://www.clinicalstudydatarequest.com/
