# Supplementary material for: Dupilumab suppresses type 2 inflammatory biomarkers across multiple atopic, allergic diseases
Source: Clin Exp Allergy. 2021 Jun 26;51(7):915–31. doi: 10.1111/cea.13954 (PMC8362102; doi:10.1111/cea.13954)
Supplement: Supplementary file 1 — Supplementary Material [file CEA-51-915-s001.pdf]

Supplementary materials for the article:

Dupilumab suppresses type 2 inflammatory biomarkers across multiple atopic, allergic diseases

Jennifer D. Hamilton; Sivan Harel; Brian N. Swanson; William Brian; Zhen Chen; Megan S. Rice; Nikhil Amin; Marius Ardeleanu; Allen Radin; Brad Shumel; Marcella Ruddy; Naimish Patel; Gianluca Pirozzi; Leda Mannent; and Neil M.H. Graham

This appendix has been provided by the authors to give readers additional information about their work.

APPENDICES

Appendix S1. Baseline demographics and history of atopic disease in patients with atopic dermatitis, asthma, chronic rhinosinusitis with nasal polyps and eosinophilic esophagitis 3

Appendix S2. Mean biomarkers at baseline and end of treatment for patients with atopic dermatitis, asthma, chronic rhinosinusitis with nasal polyps, and eosinophilic esophagitis 6

Appendix S3. The effect of dupilumab on mean (± SE) concentrations of blood eosinophils (Giga/L) 12

APPENDIX S1 Baseline demographics and history of atopic disease in patients with atopic dermatitis, asthma, chronic rhinosinusitis with nasal polyps and eosinophilic esophagitis

**Type 2 disease**	**Atopic dermatitis**		**Asthma**		**Chronic rhinosinusitis with nasal polyps**		**Atopic dermatitis**		**Eosinophilic esophagitis**	
Study name	CHRONOS		QUEST		SINUS-52		SOLO-1 and SOLO-2		EoE PoC	
Study duration	52 weeks		52 weeks		52 weeks		16 weeks		12 weeks	
Treatment group	Placebo qw + TCS, n = 315	Dupilumab 300 mg q2w + TCS, n = 106	Placebo 2mL q2w + ICS/LABA, n = 321	Dupilumab 300 mg q2w + ICS/LABA, n = 633	Placebo q2w + INCS, n = 153	Dupilumab 300 mg q2w + INCS, n = 150	Placebo qw, n = 460	Dupilumab 300 mg q2w, n = 457	Placebo qw, n = 24	Dupilumab 300 mg qw, n = 23
**Baseline demographics**										
Age, mean (SD), years	36.6 (13.0)	39.6 (14.0)	48.2 (14.7)	47.7 (15.6)	51.7 (12.7)	51.9 (11.9)	38.4 (14.0)	38.3 (14.4)	36.1 (12.8)	33.1 (8.7)
Male, n (%)	193 (61.3)	62 (58.5)	103 (32.1)	239 (37.8)	95 (62.1)	97 (64.7)	250 (54.3)	267 (58.4)	10 (41.7)	13 (56.5)

Female, n (%)	122 (38.7)	44 (41.5)	218 (67.9)	394 (62.2)	58 (37.9)	53 (35.3)	210 (45.7)	190 (41.6)	14 (58.3)	10 (43.5)
**Current history of comorbid type 2 immune diseases,[a] n (%)**										
Allergies (other than food allergy)	201 (63.8)	68 (61.8)							9 (37.5)	14 (60.9)
Allergic conjunctivitis (kerato-conjunctivitis)	68 (21.6)	31 (28.2)	49 (15.3)	89 (14.1)	10 (6.5)	6 (4.0)			3 (12.5)	3 (13.0)
Allergic rhinitis	134 (42.5)	53 (48.2)	225 (70.1)	438 (69.2)	74 (48.4)	72 (48.0)	224 (48.7)	224 (49.0)	14 (58.3)	16 (69.6)
Asthma	130 (41.3)	45 (40.9)			91 (59.5)	85 (56.7)	205 (44.6)	226 (49.5)	8 (33.3)	8 (34.8)
Atopic dermatitis	310 (98.4)	108 (98.2)	38 (11.8)	62 (9.8)	10 (6.5)	4 (2.7)	–	–	4 (16.7)	3 (13.0)
Chronic rhinosinusitis	26 (8.3)	7 (6.4)	63 (19.6)	113 (17.9)	–	–			8 (33.3)	2 (8.7)
Eosinophilic esophagitis	0	1 (0.9)	2 (0.6)	0	0	1 (0.7)			–	–
Food allergy	96 (30.5)	39 (35.5)	36 (11.2)	47 (7.4)	16 (10.5)	8 (5.3)	174 (37.8)	177 (38.7)	16 (66.7)	14 (60.9)

Hives	34 (10.8)	14 (12.7)	15 (4.7)	30 (4.7)	6 (3.9)	3 (2.0)			5 (20.8)	6 (26.1)
Nasal polyps	7 (2.2)	2 (1.8)	40 (12.5)	81 (12.8)	–	–	18 (3.9)	19 (4.2)	2 (8.3)	1 (4.3)

Note: SOLO: full analysis set; CHRONOS and EoE PoC: safety analysis set; QUEST and SINUS-52: randomized population. Grey cells indicate that data were not collected or available for these patients.

Abbreviations: EoE, eosinophilic esophagitis; ICS, inhaled corticosteroids; INCS, intranasal corticosteroids; LABA, long-acting β_2-agonist; n, number; PoC, proof of concept; qw, weekly; q2w, every 2 weeks; SD, standard deviation; TCS, topical corticosteroids.

[a]For asthma, this refers to a medical history.

APPENDIX S2 Mean biomarkers at baseline and end of treatment for patients with atopic dermatitis, asthma, chronic rhinosinusitis with nasal polyps, and eosinophilic esophagitis

**Type 2 disease**	**Atopic dermatitis**		**Asthma**		**Chronic rhinosinusitis with nasal polyps**		**Atopic dermatitis**		**Eosinophilic esophagitis**	
Study name	CHRONOS		QUEST		SINUS-52		SOLO-1 and SOLO-2		EoE PoC	
Study duration	52 weeks		52 weeks		52 weeks		16 weeks		12 weeks	
Treatment group	Placebo qw + TCS	Dupilumab 300 mg q2w + TCS	Placebo 2mL q2w + ICS/LABA	Dupilumab 300 mg q2w + ICS/LABA	Placebo q2w + INCS	Dupilumab 300 mg q2w + INCS	Placebo qw	Dupilumab 300 mg q2w	Placebo qw	Dupilumab 300 mg qw
**Serum TARC (pg/mL)**										
n	313	110	319	622	149	147	454	464	24	22
Mean (SE) at baseline	7291.72 (738.03)	9678.78 (1828.09)	398.00 (19.27)	364.66 (11.84)	370.20 (21.03)	372.27 (20.94)	5933.28 (487.89)	6864.12 (703.81)	350.83 (43.43)	275.54 (27.82)
n	120	84	248	479	127	139	194	361	19	22
Mean (SE) at end of treatment	1960.14 (377.87)	479.11 (43.13)	520.36 (143.08)	234.72 (7.95)	332.49 (20.13)	219.43 (10.77)	3239.13 (738.27)	572.33 (38.71)	322.63 (43.09)	203.38 (22.29)

n	119	84	246	473	126	137	193	360	19	21
Mean change (SE) from baseline at end of treatment	−2450.77 (592.10)	−10341.70 (2315.52)	137.66 (137.49)	−122.78 (10.02)	−15.09 (11.67)	−158.53 (14.93)	−1043.61 (446.81)	−5370.36 (700.55)	−10.11 (24.98)	−71.23 (17.90)
n	119	84	246	473	126	137	193	360	19	21
Mean % change (SE) from baseline at end of treatment	−29.14 (11.50)	−78.37 (3.97)	25.90 (9.43)	−22.74 (2.31)	1.28 (2.99)	−36.16 (1.91)	10.22 (9.49)	−69.80 (1.71)	0.14 (6.70)	−21.06 (5.73)
**Plasma eotaxin-3 (pg/mL)**										
n			318	625	150	146				
Mean (SE) at baseline			50.90 (3.25)	69.53 (19.26)	90.84 (9.08)	70.61 (3.74)				
n			246	473	127	139				
Mean (SE) at end of treatment			50.46 (2.89)	29.81 (1.73)	82.62 (8.13)	30.40 (1.28)				
n			243	469	127	136				

Mean change (SE) from baseline at end of treatment			0.28 (2.76)	−48.00 (24.33)	1.40 (4.75)	−39.95 (3.60)				
n			243	469	127	136				
Mean % change (SE) from baseline at end of treatment			57.13 (20.38)	−20.95 (5.39)	14.88 (5.42)	−39.10 (7.01)				
**Serum total IgE (IU/mL)**										
n	312	110	318	626	150	148	456	465	24	23
Mean (SE) at baseline	5082.55 (249.43)	5653.27 (423.00)	448.47 (44.67)	415.05 (28.03)	228.59 (21.94)	211.79 (21.16)	6999.88 (481.27)	6715.38 (485.50)	478.46 (198.56)	204.17 (66.31)
n	117	83	251	482	128	140	196	366	19	23
Mean (SE) at end of treatment	4203.74 (390.15)	2132.49 (331.55)	432.44 (49.46)	135.61 (14.64)	292.03 (46.24)	54.64 (5.78)	5676.28 (649.71)	3444.88 (295.04)	423.05 (169.52)	137.09 (40.04)
n	116	83	248	478	128	139	196	366	19	23

Mean change (SE) from baseline at end of treatment	−118.83 (219.48)	−3797.40 (399.55)	−4.03 (20.45)	−294.80 (23.76)	64.09 (32.08)	−156.92 (17.93)	366.67 (243.19)	−3027.83 (277.28)	−74.84 (76.03)	−67.09 (27.11)
n	116	83	248	478	128	139	196	366	19	23
Mean % change (SE) from baseline at end of treatment	68.42 (52.90)	−66.25 (3.17)	9.89 (4.33)	−58.43 (7.24)	20.82 (5.72)	−67.28 (1.63)	22.74 (4.25)	−41.90 (2.71)	4.9 (5.1)	−25.3 (2.8)
**Serum periostin (ng/mL)**										
n			319	629	150	147			24	22
Mean (SE) at baseline			80.94 (2.28)	78.42 (1.51)	113.00 (4.03)	109.70 (4.06)			62.16 (6.20)	61.18 (3.90)
n			253	490	120	128			18	23
Mean (SE) at end of treatment			75.52 (2.36)	59.82 (1.13)	108.11 (5.06)	66.06 (2.31)			73.18 (7.19)	52.67 (3.04)
n			251	488	120	127			18	22

Mean change (SE) from baseline at end of treatment			−5.05 (1.76)	−18.90 (1.40)	−4.73 (3.52)	−45.42 (3.91)			9.91 (5.20)	−8.25 (3.23)
n			251	488	120	127			18	22
Mean % change (SE) from baseline at end of treatment			−2.37 (1.88)	−17.80 (1.11)	0.15 (3.66)	−33.93 (2.64)			32.85 (19.31)	−8.22 (6.95)
**Blood eosinophils (Giga/L)**										
n	314	110	320	632	150	148	454	465	24	23
Mean (SE) at baseline	0.55 (0.03)	0.58 (0.06)	0.39 (0.02)	0.35 (0.02)	0.45 (0.03)	0.45 (0.03)	0.62 (0.03)	0.60 (0.03)	0.43 (0.06)	0.31 (0.04)
n	120	83	245	476	129	140	195	360	21	22
Mean (SE) at end of treatment	0.34 (0.02)	0.40 (0.04)	0.35 (0.02)	0.33 (0.02)	0.44 (0.04)	0.37 (0.03)	0.45 (0.04)	0.57 (0.04)	0.43 (0.07)	0.17 (0.02)
n	119	83	245	476	129	139	194	360	21	22

Mean change (SE) from baseline at end of treatment	−0.17 (0.03)	−0.20 (0.05)	−0.05 (0.02)	−0.01 (0.02)	0 (0.03)	−0.08 (0.04)	−0.07 (0.03)	0 (0.03)	0 (0.08)	−0.14 (0.03)
n	119	83	242	469	128	139	190	346	20	21
Mean % change (SE) from baseline at end of treatment	−10.02 (6.69)	−19.92 (7.07)	33.60 (13.26)	43.76 (13.93)	12.88 (8.85)	24.12 (18.25)	−1.49 (7.14)	12.89 (5.91)	−8.06 (11.94)	−43.71 (6.11)

Note: Grey cells indicate that data were not collected or available for these patients.

Abbreviations: EoE, eosinophilic esophagitis; IU, international units; ICS, inhaled corticosteroids; IgE, immunoglobulin E; INCS, intranasal corticosteroids; LABA, long-acting β_2-agonist; n, number; PoC, proof of concept; qw, weekly; q2w, every 2 weeks; SE, standard error; TARC, thymus and activation-regulated chemokine; TCS, topical corticosteroids.

APPENDIX S3 The effect of dupilumab on mean (± SE) concentrations of blood eosinophils (Giga/L). AD, atopic dermatitis; CRSwNP, chronic rhinosinusitis with nasal polyps; EoE, eosinophilic esophagitis; PoC, proof of concept; qw, weekly; q2w, every 2 weeks; SE, standard error.

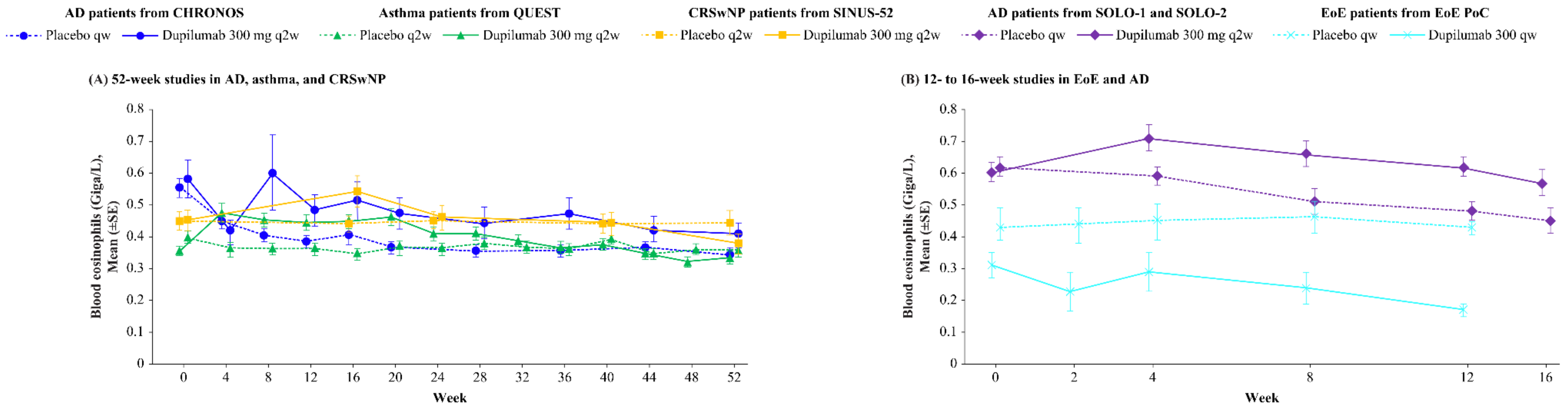

Number of patients (A)

	0	4	8	12	16	20	24	28	32	36	40	44	48	52
AD, placebo qw	314	269	240	207	187	172		151		135		125		120
AD, dupilumab 300 mg q2w	110	98	97	90	91	90		86		84		83		83
Asthma, placebo q2w	320	307	305	302	300	291	289	294	294	290	290	281	277	245
Asthma, dupilumab 300 mg q2w	632	616	587	595	596	581	577	576	560	568	561	552	537	476
CRSwNP, placebo q2w	150				143		137				131			129
CRSwNP, dupilumab 300 mg q2w	148				141		142				140			140

Number of patients (B)

	0	2	4	8	12	16
AD, placebo qw	454		324	235	208	195
AD, dupilumab 300 mg q2w	465		412	392	382	360
EoE, placebo qw	24	23	21	20	21	
EoE, dupilumab 300 mg qw	23	23	23	21	22	
